# An integrated dataset for stakeholder perceptions of environmental change and instrumented measures of change

**DOI:** 10.1016/j.dib.2018.10.112

**Published:** 2018-10-27

**Authors:** Andrew (Anaru) Kliskey, Paula Williams, Lilian (Naia) Alessa, John Abatzoglou, James Powell, Molly McCarthy, Jamie Trammell, Daniel J. Rinella

**Affiliations:** a Center for Resilient Communities, University of Idaho, 875 Perimeter Drive, Moscow, ID 83844, USA; b University of Alaska Southeast, 11120 Glacier Highway, Juneau, AK 99801, USA; c University of Alaska Anchorage, 3211 Providence Drive, Anchorage, AK 99508, USA; d Southern Oregon University, 1250 Siskiyou Boulevard, Ashland, OR 97520, USA; e United States Fish & Wildlife Service, Anchorage Fish and Wildlife Conservation Office, 4700 BLM Road, Anchorage, AK 99507, USA

## Abstract

An integrated dataset was developed that combined stakeholder perceptions of environmental change (precipitation, air temperature, water temperature, fish abundance, fish size, residential development) and comparable instrumented measures of environmental changes based on sensor records. All data were transformed to a common 3-point categorical scale to support statistical comparison of social and biophysical change for the same change variables. The integrated dataset is available on Mendeley (http://dx.doi.org/10.17632/cjfxg84bmx.1).

**Specifications table**

**Table:** 

Subject area	*Environmental change*
More specific subject area	*Perceptions of change (social), instrumented measures of change (biophysical)*
Type of data	*Tabular*
How data was acquired	*Questionnaire survey for perceptions (social data), First order weather stations for precipitation and air temperature, Water quality testing for water temperature, State sonar counts for fish abundance, Landsat imagery for residential development.*
Data format	*Transformed and standardized to 3-point nominal data for all datasets.*
Experimental factors	*Survey respondents for the social data collection were recruited from local, state, and federal agency land and water managers currently working and residing in the Southeast Alaska and Southcentral Alaska regions.*
Experimental features	*Change in biophysical phenomena was measured for each decade 1950 – 2010, and correlated with aggregated perceptions of change for each respondent for those decades during which they had resided in the region.*
Data source location	*Kenai River Watershed, Alaska (Southcentral Alaska), Juneau, Alaska (Southeast Alaska).*
Data accessibility	*Mendeley - direct URL:*http://dx.doi.org/10.17632/cjfxg84bmx.1
Related research article	*Associated research article:Williams P, Alessa L, Kliskey A et al. 2018. The role of perceptions versus instrumented data of environmental change: Responding to changing environments in Alaska. Environmental Science & Policy. DOI:*S1462-9011(17)31043-2.

**Value of the data**

• Interdisciplinary and social-ecological systems science increasingly requires integration of social and biophysical data.
• The integrated dataset described here offers a valuable approach for transforming and organizing social and biophysical data for comparison.
• Integrated datasets of social and biophysical data can support a wide ranges of analyses for comparing stakeholder perceptions of change in a phenomena with instrumented measures of change in the same phenomena.

## 1 Data

Social data include the perceptions of decision-makers from two regions of Alaska – Southcentral Alaska and Southeast Alaska. Perceptions of change for these two groups were collected for air temperature, water temperature, precipitation, fish abundance, fish size, and land use change [1]. Biophysical data include sensor records of change for the same two regions (Southcentral and Southeast Alaska). Social data were collected as categorical (nominal) data while biophysical data were transformed from ratio, interval, and ordinal data to nominal data [1].

## 2 Experimental design, materials, and methods

Social data collection used a snowballing approach for generating a list of respondents for each region – this resulted in an indicative sample of respondents not a representative sample. Respondents were administered a questionnaire survey during an interview. Human subjects ethical procedures were followed including obtaining an Institutional Review Board exemption. Biophysical data were compiled from existing US federal government and Alaska state government sensor records [1].
