# Supplementary material for: An integrated dataset for stakeholder perceptions of environmental change and instrumented measures of change
Source: Data Brief. 2018 Oct 27;21:1721–3. doi: 10.1016/j.dib.2018.10.112 (PMC6249508; doi:10.1016/j.dib.2018.10.112)
Supplement: Supplementary file 1 — Supplementary material [file mmc1.pdf]

DocuSign Envelope ID: 35B42559-15EF-4166-8CF3-EB3FC3F7AC06

AUTHOR DECLARATION

We wish to confirm that there are no known conflicts of interest associated with this publication and there has been no significant financial support for this work that could have influenced its outcome.

We confirm that the manuscript has been read and approved by all named authors and that there are no other persons who satisfied the criteria for authorship but are not listed. We further confirm that the order of authors listed in the manuscript has been approved by all of us.

We confirm that we have given due consideration to the protection of intellectual property associated with this work and that there are no impediments to publication, including the timing of publication, with respect to intellectual property. In so doing we confirm that we have followed the regulations of our institutions concerning intellectual property.

We further confirm that any aspect of the work covered in this manuscript that has involved either experimental animals or human patients has been conducted with the ethical approval of all relevant bodies and that such approvals are acknowledged within the manuscript.

We understand that the Corresponding Author is the sole contact for the Editorial process (including Editorial Manager and direct communications with the office). He/she is responsible for communicating with the other authors about progress, submissions of revisions and final approval of proofs. We confirm that we have provided a current, correct email address which is accessible by the Corresponding Author and which has been configured to accept email from: akliskey@uidaho.edu

Signed by all authors as follows:

Andrew Kliskey ____________________ November 1, 2018

Paula Williams ____________________ November 2, 2018
DocuSigned by: Paula Williams
63E8CCC401AB4C0...

Lilian Alessa ____________________ November 3, 2018
DocuSigned by:
D0D526478A0B488...

John Abatzoglou ____________________ November 1, 2018

James Powell ____________________ November 12, 2018

Jamie Trammell ____________________ November 9, 2018
DocuSigned by: E. Jamie Trammell
712AFCD800044BE...

Dan Rinella _ ____________________ November 12, 2018
DocuSigned by: Dan Rinella
B07230117A4D4AD...

Molly McCarthy ____________________ November 2, 2018
DocuSigned by: Molly McCarthy
9ED3C3678E724ED...
